# Volume-of-interest-based supervised cluster analysis for pseudo-reference region selection in [^18^F]DPA-714 PET imaging of the rat brain

**DOI:** 10.1186/s13550-018-0467-4

**Published:** 2018-12-27

**Authors:** Igor Fagner Vieira, Dieter Ory, Cindy Casteels, Fernando R. A. Lima, Koen Van Laere, Guy Bormans, Michel Koole

**Affiliations:** 1 Department of Nuclear Energy, DEN-UFPE, Recife, Brazil; 2 Nuclear Medicine and Molecular Imaging, Department of Imaging and Pathology, University Hospital and KU Leuven, Herestraat 49, B-3000 Leuven, Belgium; 3 Laboratory for Radiopharmacy, Department of Pharmaceutical and Pharmacological Sciences, KU Leuven, Leuven, Belgium

**Keywords:** Supervised clustering analysis, Pseudo-reference brain region, [^18^F]DPA-714 PET, Rat brain, Neuroinflammation

## Abstract

**Method:**

Aim of this study was to automatically select a suitable pseudo-reference brain region for the accurate, non-invasive quantification of neuroinflammation in a rat brain using dynamic [^18^F]DPA-714 PET imaging.

**Procedures:**

A supervised clustering analysis approach considering three kinetic classes (SVCA3) was used to select an appropriate pseudo-reference brain region. This pseudo-reference region was determined by selecting only brain regions with low specific tracer uptake (SVCA3_low_) or by taking into account all brain regions and weighting each brain region with the corresponding fraction of low specific binding (SVCA3_wlow_). Both SVCA3 approaches were evaluated in an animal model of neuro-inflammation induced by lipopolysaccharide injection in the right striatum of female Wistar rats. For this study setup, a population of 25 female Wistar rats received a dynamic PET scan after injection of ~ 60 MBq [^18^F]DPA-714. Animals were scanned at baseline (*n* = 3) and at different time points after inducing neuroinflammation: 1 day (*n* = 3), 3 days (*n* = 12), 7 days (*n* = 4), and 30 days (*n* = 3). Binding potential (BP) values using a simplified reference tissue model (SRTM) and the contralateral striatum as pseudo-reference region were considered as a reference method (BP_L STR_) and compared with SRTM BP values using a pseudo-reference region obtained by either the SVCA3_low_ or SVCA3_wlow_ approach for both a 90- and 120-min acquisition time interval.

**Results:**

For the right striatum, SRTM BP values using a SVCA3_low_- or SVCA3_wlow_-based pseudo-reference region demonstrated a strong and highly significant correlation with SRTM BP_L STR_ values (Spearman *r* ≥ 0.89, *p* < 0.001). For the SVCA3_low_ approach, Friedman tests revealed no significant difference with SRTM BP_L STR_ values for a 120-min acquisition time while small but signification differences were found for a 90-min acquisition time (*p* < 0.05). For the SVCA3_wlow_ approach, highly signification differences (*p* < 0.001) were found with SRTM BP_L STR_ values for both a 90- and 120-min acquisition time interval.

**Conclusions:**

A SVCA3 approach using three kinetic classes allowed the automatic selection of pseudo-reference brain regions with low specific tracer binding for accurate and non-invasive quantification of rat brain PET imaging using [^18^F]DPA-714. A shorter acquisition time interval of 90 min can be considered with only limited impact on the SVCA3-based selection of the pseudo-reference brain regions.

## Introduction

PET imaging of neuroinflammation has proven to be a valuable tool for studying and quantifying microglial activation in brain tissue associated with both healthy aging and neurodegenerative disorders such as Alzheimer’s disease (AD), Parkinson’s disease (PD), amyotrophic lateral sclerosis (ALS), and Huntington’s disease (HD) [1–3]. As an imaging biomarker, the translocator protein 18 kDa (TSPO), formerly known as peripheral benzodiazepine receptor (PBR), is a well-established target for PET imaging of neuroinflammation; since under healthy conditions, TSPO expression in the central nervous system is low while in response of neuronal insults upregulation is mainly induced in microglia, astrocytes, and endothelial cells. The most frequently used TSPO PET ligand is (R)-[^11^C]PK11195 which has several limitations in terms of high non-specific binding and poor signal to noise ratio. Therefore, other TSPO PET ligands have been developed such as [^18^F]DPA-714 which presented lower non-specific binding and higher affinity for TSPO compared to (R)-[^11^C]PK11195 [4]. [^18^F]DPA-714 has been used for clinical PET studies including both healthy volunteers and patients suffering from neurodegenerative diseases such as AD and ALS [5, 6]. In terms of PET quantification, a reference tissue model is less invasive, less prone to errors, and logistically less challenging compared to plasma input models as it avoids the need for arterial blood sampling. As such, this approach is better suited for routine practice and for longitudinal PET studies. However, in case of pathology-induced neuroinflammation, the pattern of microglial activation is generally unknown and therefore extracting an appropriate reference brain region without additional pathological or histological information may be challenging. A supervised clustering algorithm, called supervised clustering analysis (SVCA), has been proposed to automatically extract reference tissue time activity curves (TACs) from dynamic brain PET scans. This SVCA approach has mainly been developed and applied for PET imaging of neuroinflammation with (R)-[^11^C]PK11195, although a similar approach was successfully applied for other tracers such as [^11^C]PIB [7], [^11^C]TMSX [8], and [^11^C]PE2I [9]. In short, it classifies PET voxels based on the assumption that each voxel TAC is a weighted sum of normalized TACs representing different, predefined kinetic classes. Next, the value of each PET voxel is multiplied with the corresponding SVCA weight representing the fraction of non-displaceable tracer binding and the reference tissue TAC is approximated by the sum of weighted TACs of all PET voxels with the sum of weights normalized to one. Specifically for human (R)-[^11^C]PK11195 PET imaging of neuroinflammation, the number of predefined kinetic classes ranged from ten [10] over nine [11] and six [12] to four classes [13]. The latter SVCA4 approach uses predefined kinetic classes for activity in the blood pool, white matter uptake, gray matter uptake with low high specific binding, and gray matter uptake with high specific binding respectively. A SVCA4 based pseudo-reference tissue approach was also considered for human [^18^F]DPA-714 imaging of neuroinflammation [14]. Instead of applying appropriate weighting and including all PET voxels, this approach used a minimal threshold for the SVCA weights representing the fraction of non-displaceable tracer binding and took into account only PET voxels with low specific uptake. As such, it showed a high correlation with the validated two-tissue compartment plasma input model while being more robust and more accurate than a reference tissue model using only the cerebellar gray matter as reference tissue. In terms of preclinical SVCA for dynamic [^18^F]DPA-714 PET imaging of neuroinflammation in the rat brain, Sridharan et al. [15] evaluated a SVCA implementation with three kinetic classes representing “activated tissue,” “normal tissue,” and “tissue with intermediate binding,” respectively. However, they only considered a SVCA approach which included the TACs of all PET voxels with appropriate SVCA weighting to generate a reference tissue TAC.

Therefore, the primary aim of this study was to further evaluate the SVCA approach for non-invasive, simplified, and robust quantification and longitudinal monitoring of neuroinflammation in a rat model using [^18^F]DPA-714 PET imaging. For the validation, we used a rat model with local, unilateral neuroinflammation in the right striatum induced by intracerebral and unilateral stereotactic injection of lipopolysaccharide (LPS). For this animal model, quantification of [^18^F]DPA-714 PET imaging using a simplified reference tissue model (SRTM) and the contralateral region as reference tissue has been validated for monitoring local neuroinflammation [15–17] and can therefore be used as reference method for the evaluation of the SVCA approach. The secondary aim of this study was to evaluate the impact of a reduced acquisition time on the performance of the SVCA approach and on the accuracy of the resulting quantitative parameters.

## Materials and methods

### [^18^F]DPA-714 synthesis

The radiotracer [^18^F]DPA-714 was synthesized as previously described [18] with some small modifications: most importantly, the semi-preparative HPLC (high-performance liquid chromatography) purification was performed using an ethanol-based mobile phase, EtOH:NH_4_OAc 10 mM pH 7 35:65 *V*/*V*. The final preparation containing less than 10% of EtOH was sterile filtered through a 0.22-μm membrane filter (Millex®-GV, Millipore, Billerica, USA). This resulted in [^18^F]DPA-714 of > 98% radiochemical purity, with a 45–60% yield (non-decay corrected relative to starting [^18^F]F^−^radioactivity). The specific activity at end of synthesis ranged from 56 to 251 GBq/μmol. The precursor and reference compound were kindly provided by Prof. Michael Kassiou (University of Sydney, Australia).

### Animal model

Twenty-five female Wistar rats (weight 189–330 g, age 2–3 months) were used for the study. The animals were housed in groups of two, at an average temperature of 22 °C and a 12-h light/dark cycle. Food and water were given ad libitum. Three rats were used for baseline PET scanning while the other 22 rats were scanned 1 day (1D, *n* = 3), 3 days (3D, *n* = 12), 7 days (7D, *n* = 4), and 30 days (30D, *n* = 3) after inducing neuroinflammation [16]. All animals were sacrificed after PET scanning. The animals scanned 3 days after inducing neuroinflammation, consisted of two groups. For one group (3DA, *n* = 7), dynamic PET imaging was combined with arterial blood sampling to validate modeling of [^18^F]DPA-714 kinetics [17] while another group of animals (3 dB, *n* = 5) was scanned for longitudinal monitoring of neuro inflammation [16].

To induce neuroinflammation, stereotactic surgery was performed under ketamine (60 mg/kg intraperitoneal (IP)) and medetomidine (0.4 mg/kg IP) anesthesia using aseptic procedures. All animals were positioned in a stereotactic head frame (Stoelting, Wood Dale, IL, USA). A small hole was drilled in the skull at the appropriate location using Bregma as reference. Neuroinflammation was induced by injecting 50 μg of lipopolysaccharide (LPS; *E. coli* 055:B5; Sigma Aldrich, St. Louis, MO, USA) in 4 μl of sterile 0.9% NaCl solution into the right striatum at the following coordinates: 0.5 mm antero-posterior, 3 mm lateral, 5.5/4.5 mm dorsoventral. After injection of 2 μl, the needle was retracted for 1 mm dorsoventrally and another 2 μl was injected. The needle was left in place for an additional 10 min before being slowly withdrawn from the brain. The contralateral side was injected as a control with 4 μl of sterile 0.9% NaCl solution using an identical procedure.

### MicroPET imaging

PET imaging was performed on a lutetium oxyorthosilicate detector-based tomograph (microPET FOCUS-220; Siemens Medical Solutions, Malvern, PA) which has a nominative transaxial resolution of 1.35 mm full-width at half-maximum in the center of the field of view [19]. For dynamic PET imaging without arterial blood sampling, we used a multiple animal setup to allow the simultaneous acquisition of three rat brains. As such, animals were positioned off center, achieving a volumetric resolution of approximately 8 mm^3^ [20]. Data were acquired in a 128 × 128 × 95 matrix with a pixel width of 0.475 mm and a slice thickness of 0.796 mm. The coincidence window width was set at 6 ns.

Rats were injected with about 60 MBq of [^18^F]DPA-714 via a tail vein. During scanning, animals were kept under gas anesthesia (2.5% isoflurane in O_2_ at a flow rate of 1 L/min). Dynamic PET scans were acquired for 120 min in listmode. Acquisition data were then Fourier rebinned in 27 time frames (4 × 15 s, 4 × 60s, 5 × 180 s, 8 × 300 s, 6 × 600 s) and reconstructed using maximum a posteriori iterative reconstruction.

For the 7 animals of the 3DA group, dynamic PET imaging was combined with arterial blood sampling. For this purpose, an arterial cannula was placed in the femoral artery. After slow bolus injection of [^18^F]DPA-714, arterial blood was collected continuously for 1 min (15 samples, approximately one sample of 40 μl every 4 s allowing detailed (peak) blood input function with high time resolution), followed by 100-μl samples at 90, 120, 150 s and 3, 5, 10, 40, 60, 120, and 180 min. To prevent possible effects of blood volume changes and to flush the cannula, 100 μl of saline was reinjected for the 90-min sample and all subsequent samples. All blood samples were immediately stored on ice to stop tracer metabolism. The plasma time–activity curve was corrected for the fraction of unchanged radioligand that was quantified using reverse-phase (RP) HPLC (LaChrom Elite HPLC system; Hitachi, Darmstadt, Germany; Chromolith C18, 3 mm × 100 mm; Merck, MA).

### Supervised cluster analysis for preclinical [^18^F]DPA-714 brain PET

For this study, the SVCA approach for preclinical [^18^F]DPA-714 PET scanning of rat brains was limited to a volume-of-interest (VOI)-based approach and aimed at classifying rodent brain VOIs as either a pseudo-reference brain VOI or a brain VOI affected by neuroinflammation. For this purpose, spatial normalization of the dynamic PET data was done manually by adjusting translation, rotation, and scaling parameters while visually assessing the alignment to a T2-weighted rat brain template [21]. This way, the VOIs predefined on the template were projected onto the PET dynamic data to generate the corresponding TACs.

A SVCA3 approach was proposed which took into account three separate, predefined kinetic classes: one kinetic class representing brain tissue with low specific tracer binding (*K*_low_), one kinetic class representing brain tissue with high specific tracer binding (*K*_high_), and one kinetic class representing extra cerebral tracer signal (*K*_ext_).

Consequently, this SVCA3 approach was formalized as follows:

1 $$ {\mathrm{TAC}}_{\mathrm{VOI}}^n={w}_{\mathrm{low},\mathrm{VOI}}{\overline{\mathrm{TAC}}}_{\mathrm{low}}^n+{w}_{\mathrm{high},\mathrm{VOI}}\ {\overline{\mathrm{TAC}}}_{\mathrm{high}}^n+{w}_{\operatorname{ext},\mathrm{VOI}}\ {\mathrm{TAC}}_{\mathrm{ext}}^n $$

In Eq. (1), ${\mathrm{TAC}}_{\mathrm{VOI}}^n$ is the normalized TAC of a specific brain region while ${\mathrm{TAC}}_{\mathrm{ext}}^n$ represents the extra cerebral kinetic class *K*_ext_. ${\overline{\mathrm{TAC}}}_{\mathrm{low}}^n$and ${\overline{\mathrm{TAC}}}_{\mathrm{high}}^n$ are the representative normalized TACs of the kinetic classes *K*_low_ and *K*_high_. The parameters *w*_low, VOI_, *w*_high, VOI_, and *w*_ext, VOI_ are the respective weight factors of the three different kinetic classes, estimated for each brain region by a regression procedure while constraining all weights to positive values and normalizing their sum to one.

To generate normalized TACs for the different brain VOIs, the mean uptake value of each frame of the dynamic PET scan was subtracted from all voxels within that frame after which all voxels within that frame were divided by the standard deviation of uptake values of the corresponding frame. The mean uptake value and standard deviation of uptake values of each frame were determined by taking into account only voxels within a predefined brain mask, corresponding to the brain template.

Since TAC normalization was performed on a voxel level, the normalized TAC for a VOI of *N* voxels (Eq. 1) can also be expressed as:

2 $$ {\mathrm{TAC}}_{\mathrm{VOI}}^n={\sum}_N\frac{{\mathrm{TAC}}_i^n}{N}={\overline{\mathrm{TAC}}}_{\mathrm{low}}^n{\sum}_N\frac{w_{\mathrm{low},i}}{N}+{\overline{\mathrm{TAC}}}_{\mathrm{high}}^n{\sum}_N\frac{w_{\mathrm{high},i}}{N}+{\mathrm{TAC}}_{\mathrm{ext}}^n{\sum}_N\frac{w_{\operatorname{ext},i}}{N} $$

Therefore, the weights of the different kinetic classes on a VOI level are identical to the averages of the corresponding weights of all voxels included in the VOI. For our VOI-based approach, normalized TACs were generated for 16 brain regions using a predefined VOI map: the right and left striatum, cerebellum, right and left frontal cortex, right and left temporal cortex, right and left sensor motor cortex, right and left hippocampus, right and left thalamus, right and left hypothalamus, and midbrain. Next to the these brain regions, a brain mask for extra-cerebral PET signal was generated by dilating the brain mask corresponding to the brain template (IDL 8.4 dilate function with 2.0 × 2.0 × 2.0 mm structuring element) and subtracting this dilated brain mask from the non-dilated brain mask (“exBrain” mask). On the other hand, the brain mask corresponding to the brain template was eroded (IDL 8.4 erode function with 4.0 × 4.0 × 4.0 mm structuring element) to generate a brain mask with minimal contribution of the PET signal from extra cerebral bone and soft tissue regions, surrounding the brain (“inBrain” mask).

Next, ${\overline{\mathrm{TAC}}}_{\mathrm{low}}^n$ was obtained as the average normalized TAC of the three normalized, dynamic PET scans prior to LPS injection by applying the “inBrain” mask. For all animals except for the animals scanned at day 3 after LPS injection, ${\overline{\mathrm{TAC}}}_{\mathrm{high}}^n$ was determined as the average of the normalized, right striatal TAC of all 12 normalized, dynamic PET scans at day 3 after LPS injection. In order to allow an objective analysis of the dynamic PET scans at day 3 after LPS injection, the normalized, right striatal TAC of the 3DA group, consisting of 7 animals with combined dynamic PET imaging and arterial blood sampling, was used as ${\overline{\mathrm{TAC}}}_{\mathrm{high}}^n$ for the analysis of the 3 dB group, consisting of 5 animals with only dynamic PET imaging and vice versa. ${\mathrm{TAC}}_{\mathrm{ext}}^n$ was determined on an individual basis by applying the “exBrain” mask to the normalized dynamic data of each animal separately.

Since 90 min dynamic PET data also provided stable BP_ND_ estimates for the quantification of the [^18^F]DPA-714 binding using SRTM with the contralateral striatum as reference tissue, the regional SVCA3 weights of the kinetic class *K*_low_ (*w*_low_) were compared for a 90- and 120-min acquisition time interval. For this purpose, a repeated measures two-way ANOVA with Bonferroni post hoc tests was performed with a significance level of 0.05 (SPSS version 24.0).

Two different strategies were evaluated to determine the pseudo-reference TAC (TAC^pREF^). In a first approach, a ${\mathrm{TAC}}_{\mathrm{wlow}}^{\mathrm{pREF}}$was calculated as the average of the non-normalized TACs of the 16 different brain regions weighted by their corresponding SVCA3 weighting factor *w*_low, *i*_ and volume *v*_*i*_:

3 $$ {\mathrm{TAC}}_{\mathrm{wlow}}^{\mathrm{pREF}}=\frac{\sum_{i=0}^{16}{w}_{\mathrm{low},i}{v}_i{\mathrm{TAC}}_i}{\sum_{i=0}^{16}{w}_{\mathrm{low},i}{v}_i} $$

This strategy corresponds to a virtual pseudo-reference region approach and is in line with the approach presented for human (R)-[^11^C]PK11195 PET imaging of neuroinflammation [12, 13]. The second approach considered only brain regions with a weighting factor *w*_low, *i*_ higher than 0.95, similar to the approach presented for human [^18^F]DPA-714 PET imaging of neuroinflammation [14]. As such, only brain regions with low specific tracer uptake were taken into account to define an animal specific, composite pseudo-reference region. The corresponding ${\mathrm{TAC}}_{\mathrm{low}}^{\mathrm{pREF}}$was calculated as the average TAC of these brain regions weighted by the corresponding volume *v*_*i*_:

4 $$ {\mathrm{TAC}}_{\mathrm{low}}^{\mathrm{pREF}}=\frac{\sum_{w_{\mathrm{low},i}>0.95}{v}_i{\mathrm{TAC}}_i}{\sum_{w_{\mathrm{low},i}>0.95}{v}_i} $$

Right striatal binding potential values obtained with SRTM and using ${\mathrm{TAC}}_{\mathrm{L}\ \mathrm{STR}}^{\mathrm{REF}}$ of the left striatum as reference TAC (BP_*L* Str_) were compared with SRTM BP_wlow_, and BP_low_ of the right striatum calculated with ${\mathrm{TAC}}_{\mathrm{wlow}}^{\mathrm{pREF}}$ and ${\mathrm{TAC}}_{\mathrm{low}}^{\mathrm{pREF}}$ as pseudo-reference TAC respectively. For this purpose, a non-parametric repeated measures one-way ANOVA (Friedman test) with Dunn’s multiple comparison post hoc tests was performed, together with a correlation analysis and linear regression analysis (Prism GraphPad version 5.01). For all statistical tests, a significance level of 0.05 was used.

## Results

### Normalized kinetics and kinetic classes for preclinical [^18^F]DPA-714 brain PET

**Fig. 1:**
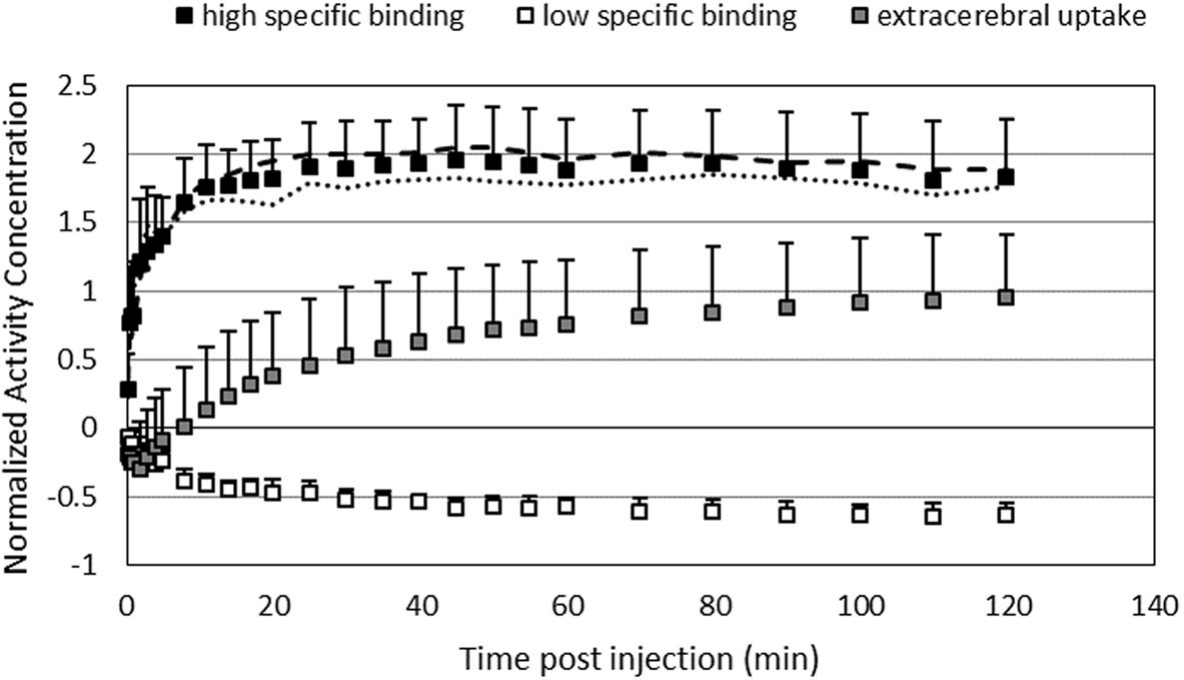
Average normalized time activity curves for the different kinetic classes representing high specific uptake (*n* = 12), low specific uptake (*n* = 3), and extra cerebral uptake (*n* = 25). Next to the normalized time activity curve for high specific uptake averaged over all 12 dynamic [^18^F]DPA-714 PET scans at day 3 after LPS injection, the average normalized time activity curve for high specific uptake is presented for the two groups of animals scans at day 3, a first group of 7 animals with combined dynamic [^18^F]DPA-714 PET scanning and arterial blood sampling (dashed line) and a second group of 5 animals with only dynamic [^18^F]DPA-714 PET imaging (dotted line)

In terms of normalized kinetics, Figure 1 presents the average, normalized TACs, ${\overline{\mathrm{TAC}}}_{\mathrm{low}}^n$, ${\overline{\mathrm{TAC}}}_{\mathrm{high}}^n$, and ${\overline{\mathrm{TAC}}}_{\mathrm{ext}}^n$, for the different kinetic classes *K*_low_, *K*_high_, and *K*_ext_ with ${\mathrm{TAC}}_{\mathrm{ext}}^n$ being determined on an individual basis. Two${\overline{\mathrm{TAC}}}_{\mathrm{high}}^n$ curves, determined for two different groups of animals scans at day 3 after LPS injection, were also presented to demonstrate the excellent reproducibility of the normalized${\overline{\mathrm{TAC}}}_{\mathrm{high}}^n$ for high specific binding. More specifically, these two groups were a group of 7 animals with combined dynamic PET imaging and arterial blood sampling and a group of 5 animals with only dynamic PET imaging.

**Fig. 2:**
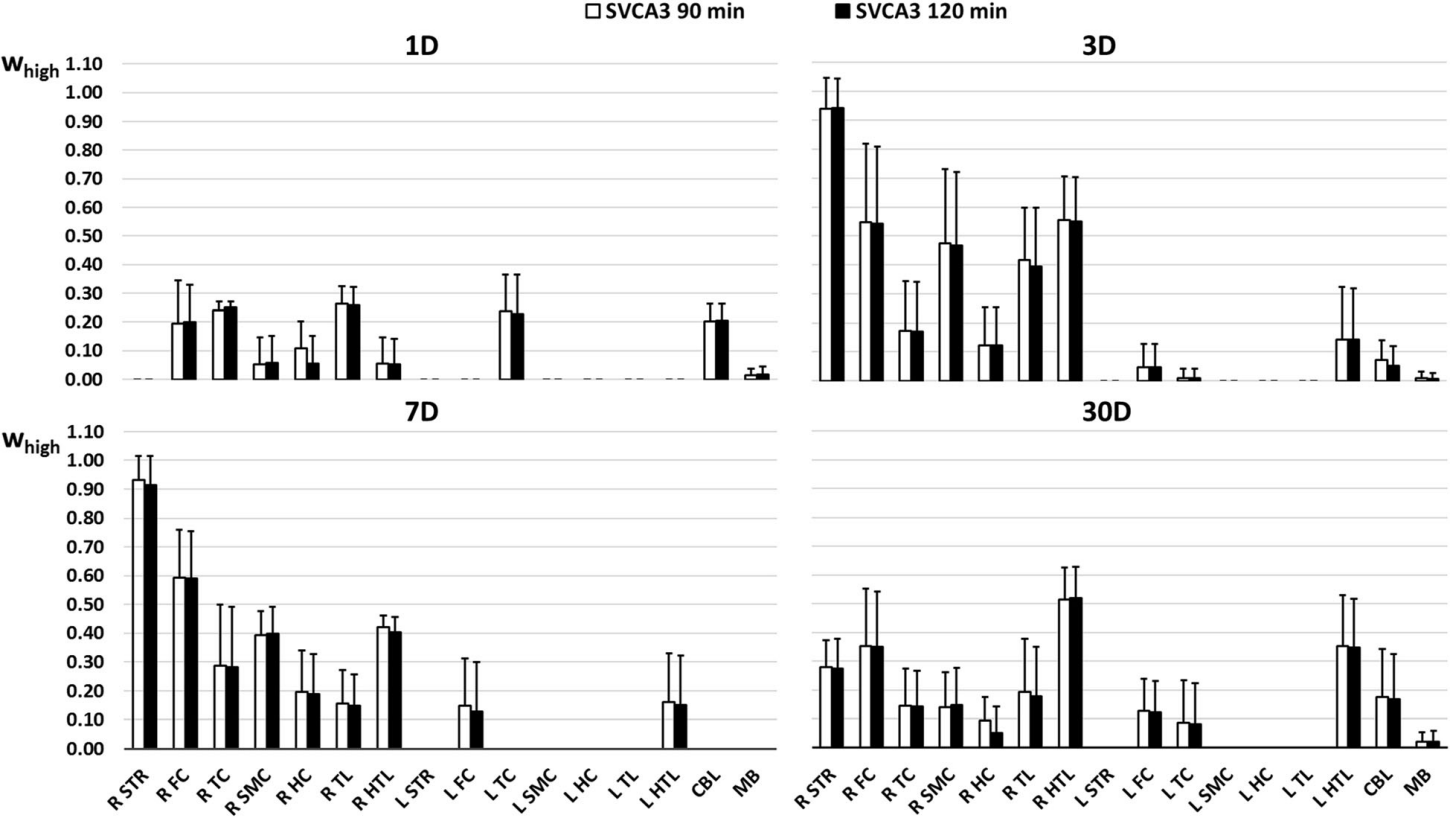
Overview of the average tissue weighting coefficients *w*_high_ of the kinetic class representing high specific tracer uptake of [^18^F]DPA-714 in a rat brain at 1 day (1D, *n* = 3), 3 days (3D, *n* = 12), 7 days (7D, *n* = 4), and 30 days (30D, *n* = 3) after LPS injection in the right striatum. Values are presented for both a 90-min and 120-min acquisition and for the right and left striatum (R-L STR, right and left frontal cortex (R-L FC), right and left temporal cortex (R-L TC), right and left sensor motor cortex (R-L SMC), right and left hippocampus (R-L HC), right and left thalamus (R-L TL), right and left hypothalamus (R-L HTL)), cerebellum (CBL), and midbrain (MB)

**Fig. 3:**
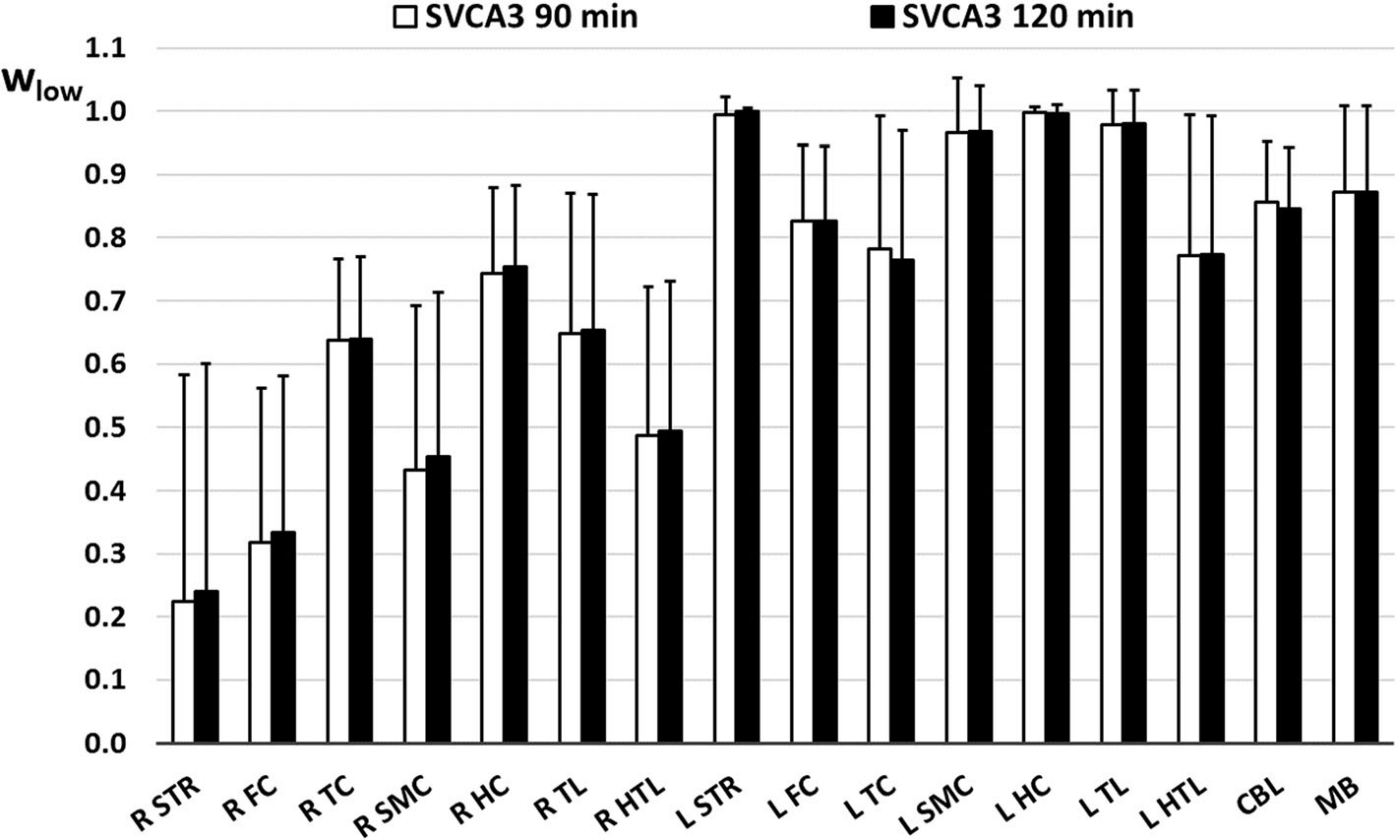
Overview of the average tissue weighting coefficients *w*_low_ of the kinetic class representing the low specific binding of [^18^F]DPA-714 in healthy rat brain tissue, averaged over various time points (*n* = 22) after LPS injection in the right striatum. Values are presented for both a 90-min and 120-min acquisition and for the right and left striatum (R-L STR), cerebellum (CBL), right and left frontal cortex (R-L FC), right and left temporal cortex (R-L TC), right and left sensor motor cortex (R-L SMC), right and left hippocampus (R-L HC), right and left thalamus (R-L TL), right and left hypothalamus (R-L HTL), and midbrain (MB)

In Figure 2, average SVCA3 weighting coefficients *w*_high_ determined for both a 90- and 120-min acquisition time interval are presented for the different time points after LPS injection (day 1, (*n* = 3), day 3 (*n* = 12), day 7 (*n* = 4), and day 30 (*n* = 3)), demonstrating the laterality of the induced inflammation and the extensive inflammation in the right striatum which picks up at day 1, peaks at the day 3, and gradually decreases at day 7 and at day 30 after LPS injection. Figure 3 presents the resulting average weights *w*_low_ for the kinetic class *K*_low_ using SVCA3 and a 90-min and 120-min acquisition time interval. Repeated measures two-way ANOVA with Bonferroni multiple comparison post hoc tests demonstrated no significant interaction between brain regions and a reduction in acquisition time for the *w*_low_ tissue weights and revealed no significant differences between the regional *w*_low_ tissue weights using a 90- and 120-min acquisition time interval.

**Fig. 4:**
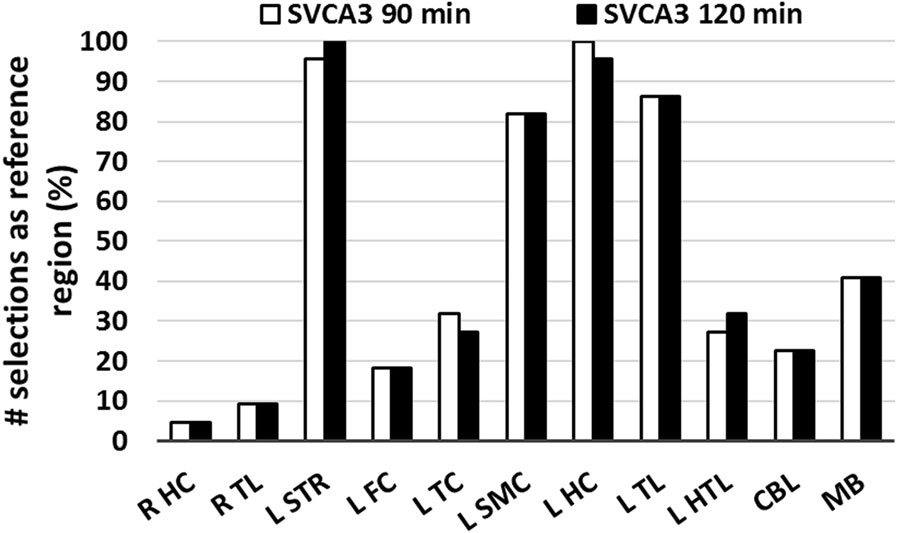
The number of selections as pseudo-reference region or region representing low specific binding (tissue weight *w*_low_ of the kinetic class representing the low specific binding greater than 0.95) considering 22 [^18^F]DPA-714 brain microPET scans at different time points after LPS injection in the right striatum (right thalamus (R TL R), left striatum (L ST), left frontal cortex (FC L), left temporal cortex (TC L), left sensor motor cortex (L SMC), left hippocampus (L HC), left thalamus (L TL), left hypothalamus (L HTL), cerebellum (CBL), and midbrain (MB)). Brain regions which were not selected as reference region are not mentioned

In terms of animal specific brain regions representing low specific binding (individual, regional tissue weighting factors *w*_low_ greater than 0.95), the number of selections of a brain region as pseudo-reference region for the different [^18^F]DPA-714 microPET brain scans is presented in Figure 4 for both a 90- and 120-min acquisition time interval while brain regions which were not selected as pseudo-reference region, were not mentioned.

**Fig. 5:**
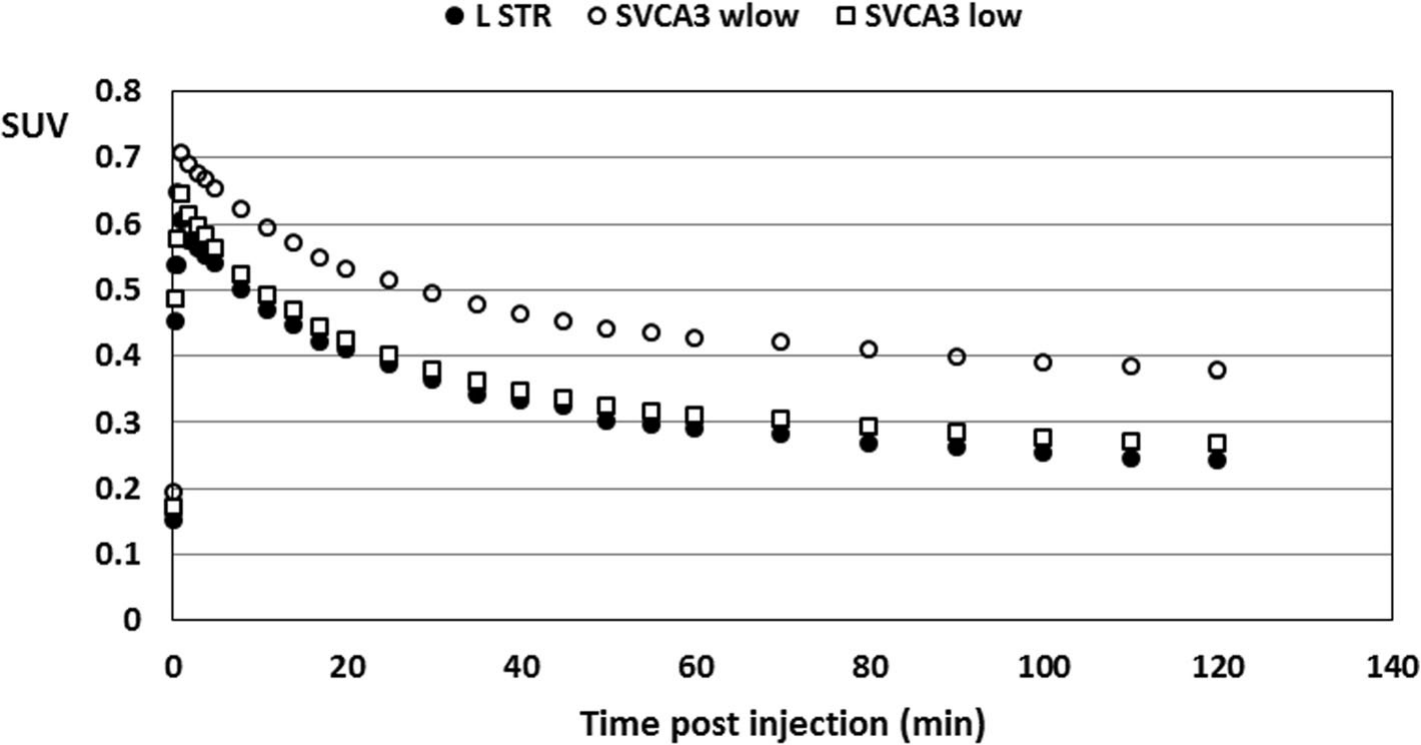
The average pseudo-reference TAC of the left striatum (L STR), together with the average pseudo-reference TAC estimated by selecting brain regions with only low specific tracer uptake (SVCA3 low) or by taking into account all brain regions and weighting each brain region with the corresponding fraction of low specific binding (SVCA3 wlow)

In order to allow a visual comparison of the resulting pseudo-reference TACs of the different SVCA approaches, Figure 5 presents the average TAC of the left striatum as reference TAC, together with the average pseudo-reference TAC estimated by selecting brain regions with only low specific tracer uptake or by taking into account all brain regions and weighting each brain region with the corresponding fraction of low specific binding.

### Quantification of the [^18^F]DPA-714 brain PET using a pseudo-reference region

**Fig. 6:**
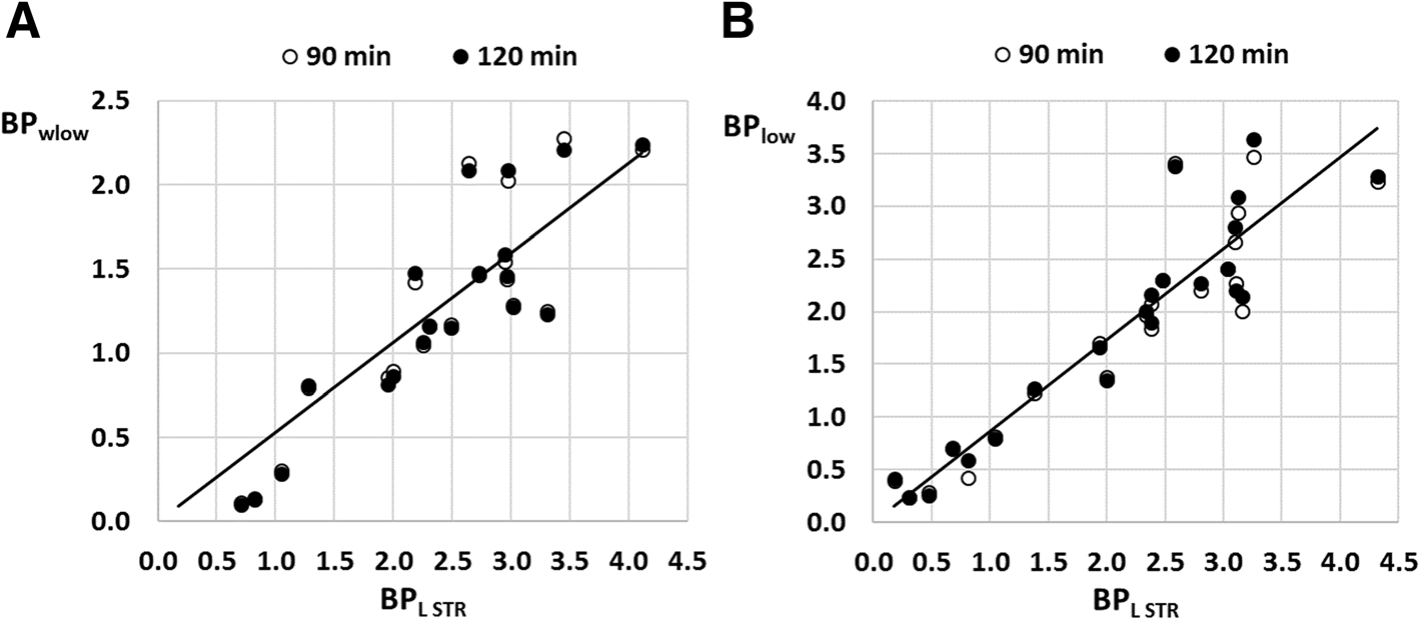
Linear correspondence between SRTM BP values for the right striatum at different time points after LPS injection using a 90- and 120-min acquisition time interval, comparing BP_L STR_ using the left striatum as reference region with BP_wlow_ using all brain regions weighted with the corresponding SVCA3 *w*_low_ weights to generate a virtual pseudo-reference region (**a**) and with BP_low_ using brain regions with SVCA3 *w*_low_ weights greater than 0.95 on an individual animal level to define a composite pseudo-reference region (**b**).The solid line represents the linear regression line (through the origin) for the 120-min acquisition time interval

Spearman correlation coefficients between BP_L STR_ and BP_wlow_, and between BP_L STR_ and BP_low_ are summarized in Table 1 for both a 90-min and 120-min acquisition time interval, together with the slope of the linear regression. All correlations were highly significant (*p* < 0.001). Non-parametric repeated measures one-way ANOVA (Friedman test) demonstrated significant differences between BP values of the different approaches. Dunn’s multiple comparison post hoc tests revealed significant differences between BP_L STR_ and BP_wlow_ for 120-min acquisition time interval (*p* < 0.001) and between BP_L STR_ and BP_low_ (*p* < 0.05) and between BP_L STR_ and BP_wlow_ (*p* < 0.001) for a 90-min acquisition time. On the other hand, no significant differences were found between BP_L STR_ and BP_low_ for 120-min acquisition time interval. Figure 6 gives an graphical overview of the correspondence between BP_L STR_ on the one hand and BP_wlow_ and BP_low_ on the other hand for the SVCA3 approach using both a 90-min and 120-min acquisition time interval.

**Table 1:** Spearman correlation and linear regression analysis between SRTM BP values for the right striatum at different time points after LPS injection using a 90- and 120-min acquisition time interval, comparing BP_L STR_ using the left striatum as reference region with BP_wlow_ using all brain regions weighted with the corresponding SVCA3 *w*_low_ weights to generate a virtual pseudo-reference region and with BP_low_ using brain regions with corresponding SVCA3 *w*_low_ weights greater than 0.95 to define a composite pseudo-reference region

	SVCA3 (90-min acquisition time)		SVCA3 (120-min acquisition time)	
BP_L STR_ vs	BP_wlow_	BP_low_	BP_wlow_	BP_low_
Spearman *r*	0.91	0.91	0.93	0.91
Slope linear regression	0.53	0.86	0.52	0.87

## Discussion

The aim of this study was to translate an SVCA approach to a preclinical setting and to enable the automatic selection of pseudo-reference brain regions with low specific [^18^F]DPA-714 binding in a rat brain. This way, non-invasive pseudo-reference tissue models can be applied for accurate quantification without prior knowledge about the spatial distribution of neuroinflammation, therefore enabling longitudinal, intra-animal monitoring of neuroinflammation and reducing or refining animal PET studies.

For human PET imaging of neuroinflammation using [^18^F]DPA-714, four kinetic classes were considered based on the dynamic PET data of healthy controls, where the blood pool class was extracted from manually segmented carotid arteries, the white matter class from white matter segmentations, the low specific binding class from cerebellar gray matter, and the high specific binding class from tracer uptake in the thalamus [14]. Since the volumetric resolution of our small animal PET system was around 8 mm^3^, it was not possible to resolve distinct gray and white matter kinetics in the rat brain. Moreover, we were unable to extract a blood pool class from the dynamic brain PET data, and extraction from the left ventricle of the heart was not possible due to field of view limitations. Therefore, we reduced and reassigned the number of classes to three kinetic classes in line with the approach presented by Sridharan et al. [15]. However, we only considered a class for low and high specific binding and did not consider an intermediate binding class, contrary to the approach of Sridharan et al., since it was hypothesized that SVCA mis-identified some “intermediate” binding voxels as normal tissue [15], leading to lower and slightly more variable BP values and thus explaining the significant difference seen when estimating BP with the SVCA approach. Instead, we included a kinetic class representing PET signal in soft tissues surrounding the brain such as the Harderian glands and in the extra cerebral bone structures due to defluorination [22–24]. Since we did not have CT data to facilitate the definition of the brain mask and the spatial normalization of the dynamic PET data [25], this extra-cerebral kinetic class allowed us to take into account small normalization errors, especially in the cortical regions as these regions are more susceptible to suboptimal alignment.

Instead of a voxel-wise approach, we opted for a VOI-based approach to define the most appropriate pseudo-reference region from a set of predefined brain VOIs. Since the weights for the different kinetic classes on a VOI level correspond to the average of the corresponding weights of all voxels included in the VOI (see Eq. 2), appropriate weighting of different TACs to define a pseudo-reference tissue TAC should be valid for both a VOI and voxel-based approach while the impact of noise is expected to be lower for a VOI-based approach. Moreover, a voxel-wise approach could require a varying, animal-specific threshold in order to select a sufficient number of voxels and obtain adequate statistics. This could be challenging, especially since only a few hundred voxels are available when taking into account the volume of a rat brain (1.4 cm^3^) and the 8 mm^3^ volume resolution of the our small animal PET system. The number of available voxels actually relates to the clinical setting by a ratio of 100 since the spatial resolution of current clinical PET systems is around 4.5 mm while the volume of the human brain is approximately 1400 cm^3^. On the other hand, considering predefined brain structures for the selection of pseudo-reference regions allowed us to define a threshold which assured the selection of at least one VOI as pseudo-reference region. However, we are aware that nowadays a better (sub-millimeter) spatial resolution and sensitivity can be achieved with the latest small-animal PET systems [26], therefore reducing the impact of noise and facilitating a voxel-wise approach.

In terms of regional classification, the time course and regional distribution of the SVCA3 weights representing brain regions with high specific uptake at different time points after LPS injection were in line with the time course and spatial distribution of neuroinflammation after LPS injection presented by previous findings [16] for both a 90- and 120-min scanning time interval. When reducing the acquisition time from 120 to 90 min, SVCA3 weights representing the fraction of brain regions with low specific uptake were not significantly different. Therefore, a SVCA3 approach using either a 90- or 120-min acquisition time interval can be used to determine which brain regions are involved in neuroinflammation and to which extent brain regions are affected by neuroinflammation.

Based on the regional *w*_low_ tissue weights obtained with SVCA3, we used two different approaches to define a pseudo-reference region. A first approach was presented for human PET imaging of neuroinflammation with (R)-[^11^C]PK11195 [13] and generated a virtual pseudo-reference region by taking into account all brain regions and weighting each region with corresponding *w*_low_ tissue weight (SVCA3_wlow_). Next, we considered a SVCA approach similar to the one presented for PET imaging of neuroinflammation in the human brain using [^18^F]DPA-714 [14]. For this approach, we determined an animal-specific, composite pseudo-reference region by taking into account only those brain regions with corresponding regional *w*_low_ tissue weights greater than 0.95 (SVCA3_low_). As such, only brain regions with low specific uptake were included in a composite regional brain mask representing a pseudo-reference region. Visual assessment of the resulting pseudo-reference TACs of both approaches (see Figure 5) clearly showed that the SVCA3_low_ approach provided a much better approximation of the left striatal reference TAC, compared to the SVCA3_wlow_ approach. Moreover, the SVCA3_wlow_ approach clearly overestimated the left striatal reference TAC, indicating that scaling the TAC of each brain region with the corresponding fraction of low specific binding does not completely eliminate the contribution of specific binding from the regional PET signal.

Next, the pseudo-reference TACs generated by the two SVCA3 approaches were used as input function for SRTM to calculate the corresponding binding potential values for the right striatum (BP_wlow_ and BP_low_ respectively). These values were compared to the binding potential values obtained with SRTM and the left striatum as reference region (BP_L STR_) since the contralateral brain region can be considered the most appropriate pseudo-reference region from a physiological point of view. While BP_wlow_ and BP_low_ demonstrated a high and significant correlation with BP_L STR_ (see Table 1), BP_wlow_ underestimated BP_L STR_ substantially while a composite pseudo-reference region including only brain regions with low specific tracer uptake substantially reduced the underestimation of BP_L STR_ to such extent that for a 120-min acquisition, no significant differences were observed between BP_L STR_ and BP_low_. These results confirmed previous findings which showed that combining all TACs with the appropriate SVCA3 weighting overestimated the pseudo-reference tissue uptake, thus causing an underestimation of BP values [15]. As such, brain regions with low specific tracer uptake are required to allow accurate quantification of neuroinflammation in a rat brain using [^18^F]DPA-714 PET imaging. On the other hand, the regional SVCA3 weights of the different kinetic classes and more specifically of the class representing non-displaceable or low specific binding can facilitate a well-founded selection of candidate pseudo-reference regions across different animals without prior knowledge about the spatial distribution of the induced neuroinflammation.

In terms of reducing the acquisition time, BP values determined by the two SVCA3 approaches were very similar for both a 90- and 120-min acquisition time interval, although a 90-min acquisition time resulted in a slightly different selection of brain regions as pseudo-reference region (see Figure 4) compared to 120-min acquisition time. Therefore, a shorter 90-min acquisition time can be considered with only very limited impact on a SVCA3-based quantification. These results are in line with previous findings that a 90-min dynamic acquisition still guaranteed an accurate quantification [17].

Main limitation of this study was the rather idealized setting where a model for acute neuroinflammation was very much confined to a small target area. For animal models of chronic neuroinflammation, the corresponding TSPO expression is expected to be more widespread across the rat brain and thus affecting more brain regions than the experimental paradigm used in this study. For these disease models, a SVCA3-based selection of a pseudo-reference region could be challenging and quantification of neuroinflammation using dynamic [^18^F]DPA-714 PET imaging and a SRTM approach could be biased. On the other hand, the SVCA3_low_ approach applied a global, minimal threshold for the regional SVCA3 weights representing low specific binding, to select animal specific pseudo-reference brain regions. The good quantitative performance of the SVCA3_low_ approach indicates that the regional SVCA3 weights representing the fraction of non-displaceable or low specific binding are comparable between animals and can be considered to some extent as a measure for the regional level of neuroinflammation or the fraction of unaffected tissue. Therefore, a SVCA3-based tissue classification can provide useful insights regarding the extent of neuroinflammation in the different brain regions and the bias that can be expected for a SVCA3-based pseudo-reference tissue approach. Moreover, in rat models with a more widespread neuroinflammation across the brain, a SVCA3 approach could facilitate the selection of brain regions with low or stable levels of neuroinflammation as pseudo-reference regions to allow the longitudinal monitoring of changes in levels of neuroinflammation in other brain regions of interest.

## Conclusion

We presented an adapted, regional SVCA3 for automatic pseudo-reference tissue selection for [^18^F]DPA-714 PET imaging of neuroinflammation in the rat brain. This SVCA3 approach considered three kinetic classes to accurately identify brain regions with low specific [^18^F]DPA-714 binding. For the quantification of the increased binding in the right striatum at different time points after LPS injection, a SVCA3-based pseudo-reference tissue model proved to be a valid alternative for the validated reference tissue model using the contralateral striatum as reference region. Therefore, this SVCA3 approach could facilitate accurate, longitudinal, and non-invasive PET quantification of neuroinflammation in the rat brain using [^18^F]DPA-714 PET. A shorter acquisition time interval of 90 min can be considered with only limited impact on the SVCA3-based selection of appropriate pseudo-reference brain regions.

## Abbreviations

AD: Alzheimer’s disease
ALS: Amyotrophic lateral sclerosis
BP: Binding potential
CBL: Cerebellum
HD: Huntington’s disease
HPLC: High-performance liquid chromatography
IP: Intraperitoneal
L FC: Left frontal cortex
L HC: Left hippocampus
L HTL: Left hypothalamus
L SMC: Left sensor motor cortex
L STR: Left striatum
L TC: Left temporal cortex
L TL: Left thalamus
LPS: Lipopolysaccharide
MB: Midbrain
PBR: Peripheral benzodiazepine receptor
PD: Parkinson’s disease
PET: Positron emission tomography
R FC: Right frontal cortex
R HC: Right hippocampus
R HTL: Right hypothalamus
R SMC: Right sensor motor cortex
R STR: Right striatum
R TC: Right temporal cortex
R TL: Right thalamus
SRTM: Simplified reference tissue model
SVCA: Supervised clustering analysis
SVCA3: Supervised clustering analysis using three kinetic classes
SVCA4: Supervised clustering analysis using four kinetic classes
TAC: Time activity curve
TSPO: Translocator protein
VOI: Volume of interest
